# c-FLIP, a Novel Biomarker for Cancer Prognosis, Immunosuppression, Alzheimer’s Disease, Chronic Obstructive Pulmonary Disease (COPD), and a Rationale Therapeutic Target

**DOI:** 10.36648/2472-1646.5.1.59

**Published:** 2019-04-26

**Authors:** Ahmad R Safa, Krzysztof Kamocki, M Reza Saadatzadeh, Khadijeh Bijangi-Vishehsaraei

**Affiliations:** 1 Department of Pharmacology and Toxicology, Indiana University School of Medicine, Indianapolis, USA; 2 Department of Neurosurgery, Indiana University School of Medicine, Indianapolis, USA; 3 Department of Pediatrics, Indiana University School of Medicine, Indianapolis, USA

**Keywords:** c-FLIP, Apoptosis, Death receptor pathways, Cancer biomarker, Antiapoptotic proteins, Cytokine resistance, Drug resistance

## Abstract

Dysregulation of c-FLIP (cellular FADD-like IL-1β-converting enzyme inhibitory protein) has been shown in several diseases including cancer, Alzheimer’s disease, and chronic obstructive pulmonary disease (COPD). c-FLIP is a critical anti-cell death protein often overexpressed in tumors and hematological malignancies and its increased expression is often associated with a poor prognosis. c-FLIP frequently exists as long (c-FLIP_L_) and short (c-FLIP_S_) isoforms, regulates its anti-cell death functions through binding to FADD (FAS associated death domain protein), an adaptor protein known to activate caspases-8 and -10 and links c-FLIP to several cell death regulating complexes including the death-inducing signaling complex (DISC) formed by various death receptors. c-FLIP also plays a critical role in necroptosis and autophagy. Furthermore, c-FLIP is able to activate several pathways involved in cytoprotection, proliferation, and survival of cancer cells through various critical signaling proteins. Additionally, c-FLIP can inhibit cell death induced by several chemotherapeutics, anti-cancer small molecule inhibitors, and ionizing radiation. Moreover, c-FLIP plays major roles in aiding the survival of immunosuppressive tumor-promoting immune cells and functions in inflammation, Alzheimer’s disease (AD), and chronic obstructive pulmonary disease (COPD). Therefore, c-FLIP can serve as a versatile biomarker for cancer prognosis, a diagnostic marker for several diseases, and an effective therapeutic target. In this article, we review the functions of c-FLIP as an anti-apoptotic protein and negative prognostic factor in human cancers, and its roles in resistance to anticancer drugs, necroptosis and autophagy, immunosuppression, Alzheimer’s disease, and COPD.

## Introduction

c-FLIP or CLARP (caspase-like apoptosis-regulatory protein) plays important roles in apoptosis, necroptotic cell death [1–4], and autophagy [1]. It also triggers resistance to anticancer agents and cytokines [1,5–10], as well as immune suppression [1,9]. Furthermore, recent results show that c-FLIP plays important roles in other diseases [11–13]. Therefore, c-FLIP is a valuable biomarker of prognosis and a reliable molecular target for developing therapeutics for cancer [1,9,14,15]. AD, and immune-related diseases. This review concentrates on the usefulness of c-FLIP as a biomarker. We discuss how c-FLIP prevents apoptosis and induces cytokine and chemotherapy drug resistance in cancer cells, its role for cancer prognosis, necrosis and autophagy, and its involvement as a marker of AD and COPD.

## Literature Review

### Apoptosis signaling pathways and role of c-FLIP

**Figure 1:**
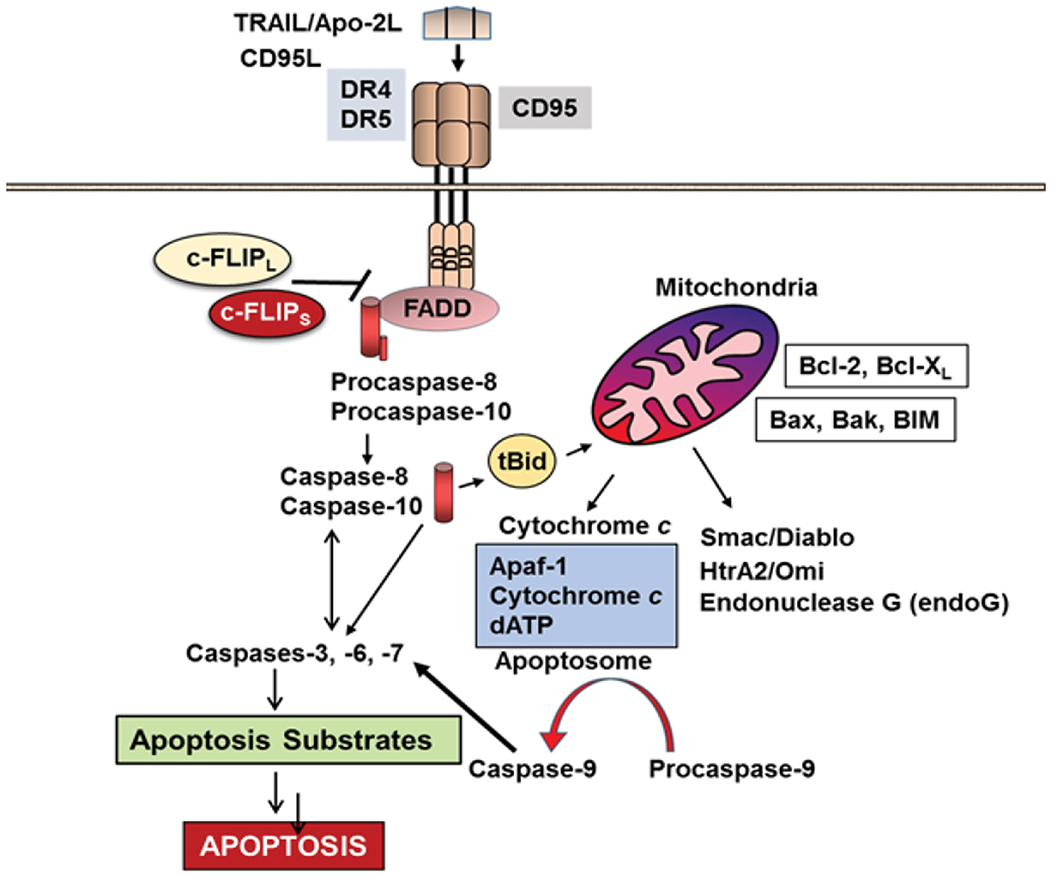
c-FLIP variants as anti-apoptotic proteins control apoptosis and pathways. Binding of TRAIL with its receptors DR4 and DR5 or interaction of Fas ligand (CD95L) to Fas receptor (CD95) triggers the death receptor (extrinsic) and subsequently mitochondrial apoptosis signaling (intrinsic) pathways through FADD-dependent autocatalytic activation of caspases-8 and -10 and Bid cleavage to truncated Bid. c-FLIP_L_ and c-FLIP_S_ isoforms suppress caspase-8 and -10 activation, therefore preventing the downstream apoptosis cascade (Modified from Safa [1]).

**Figure 2:**
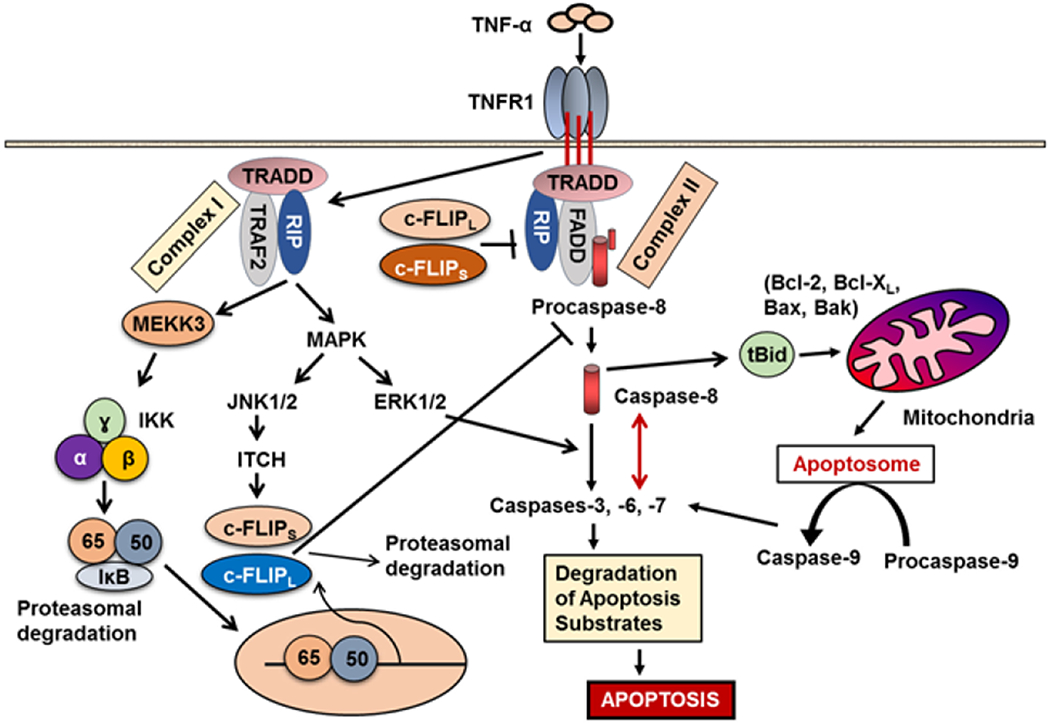
Death receptor and mitochondrial apoptosis signaling pathways and blocking TNFR1-mediated apoptosis by c-FLIP. TNF-α binds to its receptor TNFR1, which results in formation of Complex I containing TNFR1, TRADD, TRAF2 and RIP. Complex I mediates the NF-κB activation pathway and occurs through the MEKK3-IKK-IκB-NF-κB cascade, leading to the expression of c-FLIP_L_ and c-FLIP_S_ isoforms. TNF-α treatment through Complex I also activates JNK and ERK through the MAPK signaling pathway. The ubiquitin-E3-ligase ITCH promotes the ubiquitylation and proteasomal degradation of c-FLIP isoforms. As a result of degradation, levels of c-FLIP protein isoforms decrease. Complex II consists of RIP, TRADD, FADD, and procaspase-8. Caspase-8 is autoactivated and activates caspases-3 and -7, there by triggering apoptosis. Caspase-8 induces cleavage of the proapoptotic protein Bid to truncated Bid (tBid) which activates the mitochondrial apoptosis pathway that involves the release of cytochrome c and Smac/DIABLO from the mitochondria. Cytochrome c binds to Apaf1 to activate caspase-9-mediated executor caspases (Modified and updated from Safa).

Three major signaling pathways, the intrinsic or mitochondrial pathway, the extrinsic or cell surface death receptors (DRs) pathway, and the endoplasmic reticulum (ER) stress-induced apoptosis pathway are known to regulate apoptosis (Figures 1 and 2) [1,4]. In the mitochondrial apoptotic pathway, anti- and pro-apoptotic members of the Bcl-2 family cooperate and regulate the release of cytochrome c and other apoptosis-inducing factors from the mitochondria to the cytosol [16,17]. Cytochrome c and dATP bind to apoptotic proteinase-activating factor-1 (Apaf-1) and make a complex with adenine nucleotides to form the apoptosome, which promotes procaspase-9 autoactivation [17,18]. Formation of apoptosome is important in the mitochondrial apoptosis pathway and by binding to cytochrome c it activates the initiator caspase-9. This caspase in turn activates caspases-2, -3, -6, -7, -8, and -10 [17–22]. Successful apoptosis requires direct activation of the pro-apoptotic proteins Bax and Bak at the mitochondria by a member of the pro-apoptotic family of proteins including Bid, Bim, or PUMA [20]. Cytochrome c, release and opening the permeability transition pore (PTP), and a collapse of mitochondrial transmembrane potential (Δψm) are associated processes and are due to the intake of endoplasmic reticulum (ER) Ca2+ release [20–22] into cytosol.

In the death receptor (DR)-mediated or extrinsic apoptosis pathway, Fas/Fas Ligand (FasL) [CD95/CD95 Ligand, CD95L] interaction, TRAIL/DR4 or TRAIL/DR5 interaction, or the binding of tumor necrosis factor α (TNF-α) with its receptor, TNF receptor 1 (TNFR1), initiates apoptosis (Figures 1 and 2). Similarly, binding of the agonistic antibodies to these respective receptors also initiates the apoptotic signaling cascade. Following the interactions of the DR ligands or DR agonistic antibodies with their respective trimerized DRs, the adaptor molecule Fas-Associated Death Domain (FADD) is recruited to the DRs *via* death domain (DD) interactions, whereas procaspase-8, procaspase-10, and c-FLIP are recruited to the death-inducing signaling complex (DISC) *via* death effector domain (DED) interactions [23,24]. Therefore, the DISC consists of trimerized DRs, FADD, procaspase-8/-10, and c-FLIP. Procaspase-8 and procaspase-10 form a complex with FADD and are autocatalytically activated to form the active initiator caspase-8 or caspase-10.

Caspase-8 also becomes activated intrinsically, and not extrinsically, as a result of c-Myc inducing the down-regulation of c-FLIP_L_. Therefore, c-FLIP_L_ may be of importance not only in regulating the death receptor ligand-induced apoptosis, but also in apoptotic processes triggered from within the cell [25]. Moreover, chromatin immunoprecipitation (ChIP) and luciferase assays identified the binding of c-Myc to the c-FLIP_L_ promoter [26]. Moreover, after treating I/R rats with the c-Myc inhibitor 10058-F4, a significant decrease in c-FLIP_L_ and an increase in cleaved caspases-8 and 3 was seen, providing further support for the functional role of c-FLIP_L_ in intrinsic apoptosis [26].

Silencing c-FLIP caused sensitivity of tumor cells to death ligands and chemotherapeutic agents in cancer cell models [6,27]. Furthermore, in addition to its function as an anti-apoptotic protein, c-FLIP has other functions such as increased cell proliferation and tumorigenesis [3,27]. In TNF-α-triggered apoptosis (Figure 2), TNFR1 internalizes and induces formation of Complex II containing RIP, TRADD, FADD, and caspase-8. Caspase-8 autoactivation triggers activation of caspases-3 and -7, leading to apoptosis, and c-FLIP inhibits capsase-8 and -10 activation and apoptosis [3,28]. A pro-apoptotic Bcl-2 family member, Bid, is cleaved to the truncated Bid (tBid) by caspase-8 and -10 and triggers mitochondrial cytochrome c release. Thus, tBid links the death receptor pathway to mitochondrial pathway. After activation, both caspases-8 and -9 activate caspase- 3 and other caspases, and ultimately apoptosis. Mitochondria play a leading role in cellular respiration and homeostasis in the cells and transfer various signals for cell survival and death to the cytosol.

Ranjan and Pathak [29] demonstrated that c-FLIP_L_ and FADD expression participate in balancing redox potential by regulating antioxidant levels. Further, they noticed that knockdown of c-FLIP_L_ and induced expression of FADD results in rapid accumulation of intracellular ROS accompanied by JNK1 activation to enhance apoptosis. Therefore, besides their death receptor signaling, c-FLIP_L_ and FADD play important roles in preventing mitochondrial mediated apoptosis. The interaction of the TNF-α trimer to TNF receptor 1 (TNFR1) also triggers TNFR1 trimerization and Complex I formation participating in inducing the antiapoptotic proteins (Figure 2). Complex I contains TNFR1, TRADD, TRAF2, and RIP and is able to activate the NF-κB signaling pathway through the MEKK3-IKK-IκB-NF-κB cascade and subsequently activates the transcription and expression of several genes including antiapoptotic factors such as IAPs, Bcl-2, and c-FLIP [3,30]. As shown in Figure 2, TNF-α treatment through Complex I can also cause activation of JNK and ERK through the MAPK signaling pathway. The ubiquitin#specific proteases system 2 (USP2) stabilizes the ubiquitin-E3-ligase ITCH and lowers NF-κB basal activity, which leads to reduced c-FLIP mRNA production; proteasomal degradation of c- FLIP isoforms is also elevated by its negative regulator proteasome ITCH [31]. Therefore, levels of c-FLIP protein isoforms decrease and apoptosis increases. The TRAIL receptors, DR4 or DR5, can also promote alternative signaling pathways such as JNK, MAPK, or NF-κB by recruiting RIP1 and TRAF2 or TRAF5 to form a secondary signaling complex [32,33]. Activation of NF-κB in this pathway also results in increased expression of c-FLIP (Figure 2). Studies with TRADD-deficient mouse embryo fibroblasts (MEFs) have documented that RIP1 is also recruited to the TRAIL receptor by interacting with TRADD, and both RIP1 and TRADD protect against TRAIL-induced apoptosis [5,34]. In these TRADD-deficient MEFs, MAPK and NF-κB pathway activation was impaired, confirming the role of TRADD as the key adaptor protein mediating nonapoptotic signaling by DRs [5,34]. Indeed, the human T-cell leukemia virus type 1 (HTLV-1) Tax protein triggers c- FLIP production through activation of the IKK-NF-κB cascade the *via* DR4/DR5 pathway [35].

It is also known that TNF-α and Fas trigger the cleavage of mitogen-activated protein kinase/ERK kinase kinase (MEKK), resulting in production of a constitutive active form of MEKK1 and leading to JNK activation in c-FLIP knockdown cells [36]. In the absence of caspase-8 activity, the death receptors promote death by programmed necrosis (necroptosis) which requires the kinases receptor-interacting kinase 1 (RIPK1), RIPK3, and mixed-lineage kinase-like protein (MLKL) [37].

The endoplasmic reticulum (ER) stress-induced apoptosis pathway is the result of internal cellular stress like the accumulation of damaged glycoproteins [38], or exogenous stress, such as chemotherapeutic agents and hypoxia [39], which activate the process of cellular responses to stress, termed the unfolded protein response (UPR) [40]. UPR plays a major role in the homeostasis of ER. Prolonged or excessive ER stress triggers signaling pathways resulting in cell death. Triggering apoptosis *via* ER stress is usually associated with increased expression of DR5, downregulation of c-FLIP, caspase activation, and participation of the JNK pathway. ER and mitochondria cross-talk through forming mitochondria-ER associated membranes (MAMs) or mitochondria associated ER membranes (MERCs) [41] that reciprocally transmit signals under stress conditions, triggering synergistic death responses [42].

### c-FLIP spliced variants and the structure of c-FLIP isoforms

**Figure 3:**
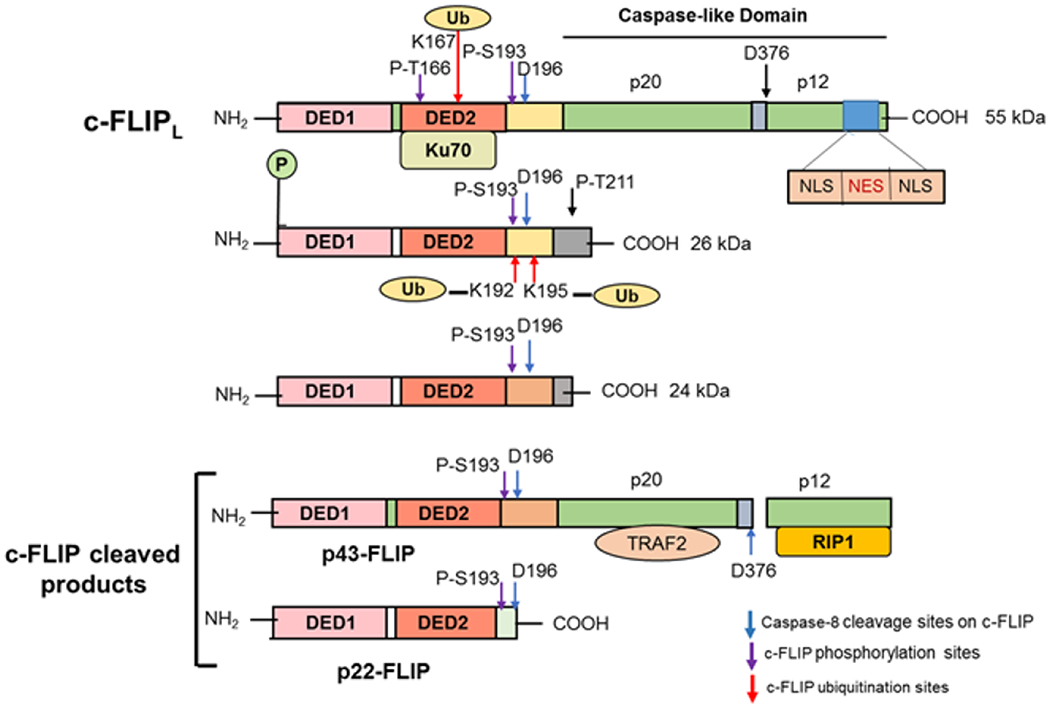
Structures of c-FLIP variants and their cleavage products. c-FLIP isoforms (c-FLIP_L_, c-FLIP_S_, and c-FLIP_R_) have two death effector domains (DED1 and DED2) at their N termini which are required for DISC recruitment. In addition to two DEDs, c-FLIP_L_ has a significant similarity to caspase-8 and has large (p20) and small (p12) caspase-like domains, which are catalytically inactive. c-FLIPS and c-FLIPR consist of two DEDs and a small C terminus. c-FLIP_L_ can be cleaved by caspase-8 generating the N-terminal fragments p43-FLIP or p22-FLIP. The phosphorylation (P) and ubiquitination (U) sites are indicated [1]. The p20/p12 regions interact with TRAF2 and RIP1, respectively, and Ku70 binds to DED2 (Modified and updated from Safa [1]).

The mammalian cellular homolog of viral FLICE-inhibitory proteins (v-FLIP_S_) was termed c-FLIP [43]. While c-FLIP consists of a family of alternatively spliced variants, three of these variants including the 26 kDa short form (c-FLIP_S_), the 55 kDa long form (c-FLIP_L_) and the 24 kDa form (c- FLIP_R_) are expressed as proteins (Figure 3) [20,44]. c-FLIP_R_ is smaller than c-FLIP_S_ and has a similar pattern of expression as c-FLIP_S_ during activation of primary human T cells and is strongly induced in T cells upon CD3/CD28 co-stimulation [45]. In humans, three isoforms of c-FLIP, c- FLIP_L_ (55 kDa), c-FLIP_S_ (27 kDa), and c-FLIP_R_ (25 kDa) have been identified [1,46]. These isoforms contain two DEDs (Figure 3). c-FLIP_L_ also contains a catalytically inactive caspase-like [44] domains (p20 and p12). Moreover, c-FLIP_S_ has an additional isoform-specific 19 amino acids in its C-terminal domain. Moreover, c-FLIP_L_ has a caspase-8 cleavage site at position Asp-376 (LEVD) and cleavage at this site results in generation of the fragment variant p43c-FLIP, p43- FLIP, and p22-FLIP [1,47]. The C-terminal region of c-FLIP_S_ and c-FLIP_R_ plays an important role in ubiquitination and degradation and their anti-apoptotic function.

### c-FLIP activates cytoprotective and proliferation pathways

As we previously discussed [1] several pathways with major roles in regulating cell survival, proliferation, and carcinogenesis can be activated by c-FLIP (Figure 2) [47–55]. DNA-PK/Akt pathway also can regulate the expression of c-FLIP [56]. Furthermore, interaction of c-FLIP_L_ with MKK7 might selectively suppress JNK activation [57]. c-FLIP may also regulate pathways participating in the production of inflammatory cytokines, tumor cell migration and metastasis [58,59], alter cell cycle progression and enhance cell proliferation and carcinogenesis [1,9]. Furthermore, overexpression of c-FLIP can alter cell cycle progression and enhance cell proliferation and carcinogenesis [1,9].

### c-FLIP as a an anti-apoptotic protein

It has been shown that c-FLIP inhibits death receptor-mediated apoptosis as well as apoptosis induced by a variety of cancer chemotherapeutic agents and small molecule targeted anticancer compounds and ionizing radiation [1,51]. Additionally, major roles for c-FLIP in promoting the survival of immunosuppressive tumor-promoting immune cells have been discovered [9]. Thus, c-FLIP is a rational anticancer therapeutic target. c-FLIP_L_ modulates signals [11] leading to both cell death and cell growth [60]. c-FLIP_L_ is upregulated in human tumors [51,61], rendering tumor cells resistant to therapies and immune surveillance [62], Regulatory T cells or Tregs turn off immune responses and have been used for immunotherapies. Treg cells compared to conventional T cells are more susceptible to apoptosis due to low c-FLIP expression [63]. Treg-specific deletion of c-FLIP in mice has been shown to cause fatal autoimmune disease [29]. Therefore, c-FLIP function is essential for cell homeostasis and prevention of autoimmunity in Treg [63]. Given the fact that Treg cells control autoimmunity and inhibit anti-cancer immunity, c-FLIP could be used as a therapeutic target to modulate Treg cell abundance and immune responses in cancer or autoimmune disease [63].

c-FLIP has been shown to affect immune regulatory pathways enriched in NF-κB response to TNF, cytokine network, and genes upregulated by IL-6 *via* STAT3 [64]. This specific gene upregulation may be partially linked to NF-κB activation induced by the nuclear translocation of c-FLIP [64]. These authors uncovered a critically important dual role of c-FLIP in myeloid cells. In mature monocytes, cancer-induced c-FLIP expression promoted immune suppressive functions and increased survival. On the other hand, constitutive c-FLIP activation in the myeloid lineage induced chronic inflammation associated with myeloproliferation and immune suppression. These results revealed that c-FLIP emerged as a novel factor for controlling cancer-associated chronic inflammation and immune dysfunction.

### Role of c-FLIP in programmed necroptosis

Necroptosis is physiologically regulated by specific proteins including RIPK1, receptor interacting protein kinase 3 (RIPK3, also known as RIP3), and mixed lineage kinase-like protein (MLKL) [1]. Receptor interacting protein kinase 1 (RIPK1), also known as RIP1, is a protein which interacts with the TNF-α receptor 1 signaling complex and recent advances have shown the critical roles of RIPK1 in cell survival, apoptosis, and necroptosis [65,66]. Necroptosis is induced as a result of inhibiting caspase-8 activation regulated by the death platform complex, Ripoptosome, and c-FLIP isoforms can switch apoptotic and necroptotic cell death. Moreover, the cellular inhibitor of apoptosis proteins (clAPs) blocks Ripotosome formation [1]. Interestingly, while c-FLIP_L_ is able to prevent Ripoptosome formation, c-FLIP_S_ promotes its formation [1]. Therefore, these c-FLIP isoforms in the Ripoptosome determine whether cell death occurs by RIP3-dependent necroptosis or caspase-dependent apoptosis [1,67,68]. Molecularly, RIPK1 phosphorylates and activates RIPK3, and activated RIPK3 then phosphorylates MLKL. Subsequently, the phosphorylated MLKL oligomer form translocates to the plasma membrane and induces necrotic cell death by forming pores on the plasma membrane [69].

### Autophagy and c-FLIP

In addition to inhibiting apoptosis, c-FLIP_L_ can affect autophagy by (1) direct action on autophagy by competing with Atg3 binding to LC3, reducing LC3 processing and inhibiting autophagosome formation [1,70,71] and (2) by interacting with procaspase-10, it forms an enzymatic complex that can cleave the Bcl-2 associated transcription factor 1 (BCLAF1), which is an autophagy inducer. This cleaved BCLAF1 form displaces Bcl-2 from an inhibitory complex with Beclin-1 and Beclin-l-induced autophagy [71]. Disrupting or preventing formation of the procaspase-10/c-FLIP_L_ complex may induce autophagic cell death [71]. Recently, it was shown that inhibition of c-FLIP overcomes acquired resistance to sorafenib by reducing endoplasmic reticulum stress (ERS)-related autophagy in hepatocellular carcinoma (HCC) [72].

### c-FLIP as a valuable prognostic biomarker in various cancers

c-FLIP variants (c-FLIP_L_ and c-FLIP_S_) serve as prognostic biomarkers for various cancer types. As shown in Figure 3, c-FLIP_L_ has a bipartite nuclear localization signal (NLS) and a nuclear export signal (NES) in its C-terminal region, required for its transport between the nucleus [10] and the cytosol [73,74]. High expression of c-FLIP is lethal in human cancers including ovarian, colon, cervical, glioblastoma, breast, colorectal, and prostate cancers, and multiple myeloma [1,50,51,61,75,76]. Collectively, c-FLIP expression is frequently upregulated in various malignancies and correlates with poor prognoses. Immunohistochemical analysis has shown two distinct pools of c-FLIP_L_, which correlate the expression and the subcellular localization of c-FLIP_L_ protein with patient therapeutic and survival outcomes [75,76]. Humphreys et al. [9] in a cohort of 184 non-small lung cancer (NSCLC) patients demonstrated high cytoplasmic but not nuclear c-FLIP significantly correlated with decreased overall survival. Valnet-Rabier et al. [77] showed that in 32 Burkitt’s lymphoma cases, a mainly cytoplasmic pool of c-FLIP was highly correlated with poor patient outcome [77]. Several studies have identified c-FLIP as an independent adverse indicator in cancer and found both c-FLIP_L_ and c-FLIP_S_ play important roles in cancer patient treatment outcomes and that the expression of particular c-FLIP isoforms has prognostic clinical value [66,78–86]. Ullenhag et al. [87] showed in colorectal (CLR) cancer patients that c-FLIP_L_ is an independent marker of poor prognosis. Furthermore, a high c-FLIP level was shown to be an independent adverse prognostic marker in stage II and III CLR cancer and might identify patients with a high possibility of relapse [78]. Moreover, c-FLIP_L_ mRNA significantly correlates with poorer overall survival of a cohort of acute myeloid leukemia (AML) patients [88]. Zang et al. [89] using immunohistochemistry found c-FLIP protein expression in IA2-IIIA cervical-squamous cell carcinoma patients and showed that high c-FLIP level was an independent negative indicator for disease-free survival (p=0.015). Lee et al. [90] found that c-FLIP_L_ expression in invasive breast carcinomas with c-FLIP_L_-positive patients showed a poor prognosis (p<0.01). Furthermore, expression of c-FLIP_L_, lymph nodes status, and molecular subtypes were independent prognostic factors for these patients (p<0.05). McCourt et al. [91] have reported that expression levels of c-FLIP and heat shock protein 27 (HSP27) in prostate cancer correlated with the Gleason score sum and pathologic stage. A prostate cancer Gleason score or grade assists in determining how aggressively the tumor is likely to behave. The score helps to classify the cancer by grading how quickly it is likely to grow and is an indicator of how likely it is to metastasize outside the prostate gland. Elevated expression of c-FLIP was shown to antagonize the therapeutic response to androgen receptor targeted therapy in castration-resistant prostate cancer (CRPC) [92]. Furthermore, the overexpression of stromal c-FLIP promotes androgen-dependent prostate cancer growth and invasion [93]. Another significant role of c-FLIP is in the carcinogenesis and aggressiveness of endometrial carcinoma and might be a critical prognostic factor in this cancer [94]. Acute human papillomavirus (HPV) infection causes cervical intraepithelial neoplasia marked by high copy episomal viral DNA and L1/L2 capsid protein expression in the cells that facilitate sexual viral transmission. Recently, Nuovo et al. [14] showed that an increased level of c-FLIP and elevated expression of importin-β, exportin-5, Mcl1, p16, and Ki67 are new biomarkers of human papillomavirus infection in acute cervical intraepithelial neoplasia.

Identification of reliable biomarkers remains a crucial factor to evaluate clinical progress in pancreatic ductal adenocarcinoma (PDAC), a lethal cancer. Haag et al. [94] found that c-FLIP overexpression in pancreatic intraepithelial neoplasia (PanIN) lesions (the most prevalent type of early lesion, arising from the ductal epithelial cells leading to moderate dysplasia, high-grade dysplasia, and invasive carcinoma), and PDAC, compared to normal pancreatic ducts. In addition, knockdown of c-FLIP increased death receptor-triggered apoptosis in PDAC cell lines. Schmid et al. [95] demonstrated that c-FLIP expression status is a [32] valuable prognostic biomarker in PDAC. Subsequently, these investigators explored the prognostic significance of c- FLIP protein expression in PDAC in a well-defined cohort including clinical parameters, and other PDAC cohorts. Interestingly, the complete lack of c-FLIP was associated with a highly aggressive clinical course [94]. c-FLIP_L_ is usually considered to function as an antiapoptotic protein [96]. However, it is also believed that c-FLIP_L_ may also function as a pro-apoptotic protein [48], depending on its isoform and expression levels [48,96]. Indeed, it is known that high [19] expression of c-FLIP_L_ blocks caspase-8 activation [27,96] while physiological levels of c-FLIP_L_ enhance oligomerization and autoproteolytic processing of caspase-8 [27,96]. c-FLIP_S_ has been implicated to function in antiapoptotic signaling as well as in increased cell death [48]. Interestingly, c-FLIP_S_ has been shown to promote necroptosis in response to Toll-like receptor 3 stimulation and depletion of IAP proteins [68] by increasing the formation of a cytosolic complex containing RIP1, FADD, and caspase-8 [1,68]. Therefore, the finding that the absence of c-FLIP is an indicator of poor prognosis in PDAC may be due to concept that c-FLIP_L_ can function both as an antiapoptotic and proapoptotic factor, depending on the tumor environment. The absence of c-FLIP in PDAC as an independent indicator of short overall survival implies that c-FLIP has prognostic value. Moreover, further research into the prognostic relevance of c-FLIP in PDAC is required to validate the impact of this protein as a prognostic biomarker for this devastating disease. Increased c-FLIP expression is a frequent event in stomach carcinoma [81,97], and as McCourt et al. [91] suggested, stomach carcinoma cells *in vivo* may require c-FLIP expression to evade apoptosis and its expression is associated with tumor cell proliferation in this cancer [98]. Additionally, c-FLIP expression and its relationship with clinicopathologic features of melanoma have been actively explored [99]. c-FLIP was found to have an important role in the aggressiveness of malignant melanoma and is a useful prognostic marker for patients with this disease [99].

### c-FLIP as a mediator of anticancer therapy resistance

Increased expression of c-FLIP in various tumor types is associated with the chemotherapeutic resistance and silencing of c-FLIP restores the proapoptotic signaling cascades efficiently to enhance chemosensitivity [1,12,46,61]. The association between expression of c-FLIP variants and poor prognosis relates to the fact that c-FLIP confers resistance to a number of anticancer agents [6,48,99–102] including sorafenib [103], the inhibitor of RAF-1 and class II receptor tyrosine kinase. c-FLIP triggers TRAIL resistance due to its increased expression level in various human tumors as well as in cancer stem cells (CSCs) from these tumors [104]. Furthermore, silencing c-FLIP expression or using c-FLIP inhibitors sensitizes tumor cells and CSCs to TRAIL and drugs like Taxol, doxorubicin, cisplatin, gemcitabine, etc. [46,100,104–106]. CSCs are also resistant to chemotherapy and radiotherapy and play a significant role in cancer recurrence [100]. Expression levels of c-FLIP isoforms were significantly higher in glioblastoma cancer stem cells (GSCs) than the entire GBM tumor cell population, and c-FLIP silencing in GSCs enhanced TRAIL and temozolomide (TMZ)-induced apoptosis [107]. It is known that breast cancer stem cells (BCSCs) mediate tumor recurrence and drive tumor metastasis [105,108]. Piggott et al. [108] reported a therapeutic approach to selectively eliminate BCSCs. They found that c-FLIP is upregulated in BCSCs from various breast cancer subtypes and that silencing of c-FLIP by its siRNA partially sensitizes these cells to the anticancer agent TRAIL. Significantly, their data demonstrated that BCSCs are sensitive to derepression of the proapoptotic pathway by TRAIL due to c-FLIP silencing, which results in an 80% reduction in primary tumors, a 98% reduction in tumor metastases, and the loss of BCSC self-renewal.

**Figure 4:**
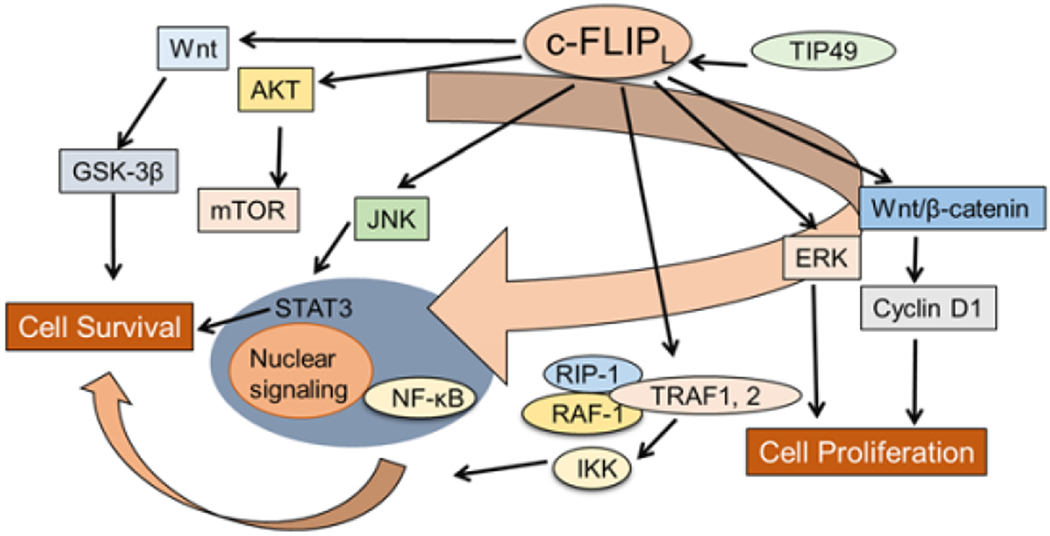
Roles of c-FLIP_L_ on cell survival and cell proliferation. As discussed in the text, in addition to its functional role in inhibiting apoptosis by binding to procaspases-8 and -10 and inhibiting their activation, c-FLIP_L_ activates cell survival and cell proliferation by controlling MAPK, AKT, mTOR, Wnt/β-catenin, NF-κB, and STAT3 signaling pathways.

Overall, c-FLIP can serve as a biomarker for detecting CSCs that are refractory to cell death, and inhibition of c-FLIP by pharmacological agents or genetic approaches may be a rational therapeutic strategy to increase the efficacy of anti-cancer agents and eliminate the apoptosis-resistant CSCs. Phenotypic plasticity is a new paradigm for understanding the origin of CSCs and the genesis of interconversion between differentiated cells and CSCs, which acquire self-renewal, proliferation, and resistance to therapy [109,110]. Recently, Thakur and Ray [111] identified NF-κB as a regulator of dedifferentiation, which increases both TNF-α and PIK3CA expression only in the enriched side-population (SP) containing CSCs, but not in the nonside-population in platinum-resistant ovarian cancer cells treated with cisplatin. Activation of PI3K/AKT signaling pathway drives SPs into an undifferentiated state, through increased c-FLIP, P21, and P27 expression [111]. Interestingly, Piggott et al. [112] recently identified a novel mechanism of acquired vulnerability to an extrinsic apoptosis stimulus in breast cancer primary cultures and BCSCs with acquired resistance to tamoxifen (TAMR) or Faslodex (fulvestrant), which has both therapeutic and prognostic potential for breast cancer therapy. In parallel with developing an endocrine resistance phenotype, these cells acquired TRAIL sensitivity, which correlated with decreased expression of intracellular levels of c-FLIP and an increase in JNK-mediated phosphorylation of the E3-ligase ITCH, which degrades c-FLIP. Furthermore, while the apoptosis inducing agent lapatinib has clinical efficacy in treating trastuzumab-refractory HER2-positive breast cancers, significant proportions of patients develop acquired resistance to the drug and develop progressive disease. Eustace et al. [113] demonstrated that the development of acquired resistance to lapatinib resulted in triggering TRAIL sensitivity. Mechanistically, increased sensitivity to TRAIL in these cells was related to decreased phosphorylation of AKT, elevated level of FOX03a and reduced expression of c-FLIP [113]. c-FLIP_L_ and c-FLIP_S_ display several roles in various cellular signaling pathways, and activate and/or upregulate several cytoprotective and pro-survival signaling proteins that include Akt, ERK, and Wnt (Figure 4). Strategies to develop new cancer therapeutics that improve the efficacy and decrease the toxicity of chemotherapeutic agents by targeting specific c-FLIP isoforms is very attractive [48]. The association between c-FLIP expression and poor prognosis in tumors may be because c-FLIP confers resistance to several anticancer agents [46,100,104,106]. We have reported that increased expression of c-FLIP_L_ or c-FLIP_S_ triggers resistance to Taxol by interacting with caspases-8 and -10 and silencing c-FLIP isoforms increases Taxol-induced apoptosis in malignant cells [6]. In colorectal cancer (CRC), high c-FLIP expression triggered resistance to #CRC standard-of-care chemotherapeutic agents, 5-fluorourcil-and oxaliplatin-triggered apoptosis both *in vitro* and *in vivo* [114]. Moreover, siRNA mediated silencing of c-FLIP isoforms (particularly c-FLIP_L_) to significantly increase cell death by these drugs. Similarly, cisplatin in NSCLC models, Taxol in leukemia [6], anticancer drugs in renal cancer cells [106] and ionizing radiation in NSCLC [114] trigger more apoptosis when c-FLIP was silenced or inhibited by pharmacological inhibitors [1,46,85,101,114]. Interestingly, high expression of cytoplasmic c-FLIP is indicative of poor prognosis [75]. Recent data also show that inhibition of the bromodomain and extra terminal domain (BET) family inhibitors effectively inhibit c-FLIP expression and sensitize KRAS-mutated NSCLC cells to pro-apoptotic agents TRAIL and cisplatin [115].

### c-FLIP and the tumor immune microenvironment

c-FLIP effects on immune effector cells is a potential reason for the correlation between c-FLIP expression and poor prognosis in tumors with high c-FLIP expression. c-FLIP can prevent cell death induced by immune effector cells [63]. In fact, cytotoxic T lymphocytes (CTLs) and natural killer cells express Fas ligand (FasL) and TRAIL, but may exert immune resistance in tumors with high c-FLIP expression. Overexpression of c-FLIP has been shown to allow establishment of tumors in immune-competent mice by blocking Fas-dependent cell death triggered by CTLs on their target tumor cells [62]. c-FLIP is also important in the survival of monocytic myeloid-derived suppressor cells (MDSCs) through its ability to inhibit death receptor mediated extrinsic apoptosis [12,62,116]. These MDSCs are immunosuppressive cells that are recruited to tumors [117] and MDSC numbers may be a marker for poor prognosis in cancer patients [118,119]. Zhao et al. [120] have shown that signaling of TNFR-2, but not TNFR-1, promoted MDSC survival through upregulation of c-FLIP. These results demonstrate that TNFR-2 signaling promotes MDSC survival and accumulation through c-FLIP overexpression and helps tumor cells evade the immune system [120]. Furthermore, c-FLIP is important for FoxP3+ regulatory T cells (Tregs) survival and maintaining local immunosuppressive environments [64]. Particularly significant is that c-FLIP knockout in dendritic cells enhanced production of TNF-α, IL2, or granulocyte-macrophage colony-stimulating factor (GM-CSF), in response to stimulation of TLR4, TLR2, and dectin-1 and increased T cell activation [121]. Therefore, c-FLIP [64] has a functional role in immunosuppression.

### c-FLIP in Alzheimer’s disease (AD)

Abnormal c-FLIP protein expression has been identified in several diseases including multiple sclerosis (MS), Alzheimer’s disease (AD), diabetes mellitus, rheumatoid arthritis (RA) and various cancers [30]. Fossati et al. [122] have fund that the TRAIL receptors DR4 and DR5 specifically mediate oligomeric amyloid-β (Aβ) induction of apoptosis in human microvascular cerebral endothelial cells through caspases-8 and -9 activation. Direct binding assays using receptor chimeras have confirmed the specific interaction of oligomeric Aβ with DR4 and DR5. DR4 and DR5 upregulation and colocalization with Aβ at the cell membrane suggests their involvement as initiators of the apoptotic cascade [122]. Aβ induced c-FLIP downregulation and the caspase-8-triggered mitochondrial pathway of apoptosis through engagement of cleaved Bid. Conversely, apoptosis protection achieved through siRNA silencing of DR4 and DR5 highlighted their active role downstream apoptotic pathways unveiling new targets in (i.e., c-FLIP, DR4, and DR5) for therapeutic intervention for AD. Chemicals and miRNAs known to upregulate [77] c-FLIP may be used to relieve depression and anxiety among AD patients. The antidepressant fluoxetine upregulates expression of c-FLIP through activating the c-FLIP promoter region spanning nucleotides −414 to −133, including the CREB and SP1 sites, and inhibits LPS-induced apoptosis in hippocampus-derived neural stem cells (NSCs) [123,124]. Moreover, fluoxetine treatment significantly inhibited the induction of proinflammatory factor IL-1β, IL-6, and TNF-α in the culture medium of LPS-treated NSCs. Therefore, the activation of c-FLIP by fluoxetine indicates its role in neuroprotection. Recent results show that fluoxetine could activate the Wnt/β-catenin signaling pathway and reduce amyloidosis in AD brain tissue [125]. Interestingly, Wnt/β-catenin is activated by c-FLIP [124]. Therefore, c-FLIP either directly or indirectly may be involved in initiating and maintaining AD. Accumulation of β-amyloid and hyperphosphorylated tau protein in AD probably triggers the neurofibrillary tangles and plaques in this disease [123]. At the present, there is no histologic marker for AD and recent evidence suggests that the hyperphosphorylation of tau protein in neurons may be [9,36] critical in AD. Previously, miR-512 has been shown to prevent c-FLIP expression and enhance Taxol triggered apoptosis in hepatocellular carcinoma cells [125]. Corroborating these results, miR-512 significantly is reduced in AD brain sections with high level of hyperphosphorylated tau protein. Interestingly, immunohistochemistry documented that c-FLIP and MCL1, the 2 targets of miRNA-512, were significantly upregulated in AD brain, were colocalized with the abnormal tau protein, and no apoptosis was noted in these areas [12]. Therefore, these results suggest that increased expression of c-FLIP and MCL1 may change the balance between apoptosis and anti-apoptotis events in neurons. Similarly, using neuronal cell cultures, Nuovo et al. [11] suggested that molecular changes including accumulation of MCL1 and c-FLIP in the affected neurons in AD prevent cell death and accumulate hyperphosphorylated tau and β-amyloid. There is no authentic histologic marker for AD and the above data underscore the significance of MCL1 and c-FLIP as biomarkers for this disease and may offer clues to its etiology. Strategies to upregulate these proteins in Alzheimer’s disease may help identify agents to prevent or inhibit the progress of this severe the disease.

### c-FLIP as a biomarker of COPD

Cigarette smoking (CS) is a critical risk factor for COPD [125]. Pouwels et al. [126] found the c-FLIP gene, CFLAR, is has a relationship with CS-triggered release of damage-associated molecular patterns (DAMP) in airway epithelial cells. Recently, Faiz et al. [13] studied the effect of CS on CFLAR expression levels in bronchial biopsies from smokers and non-smokers and isoform-expression of CFLAR transcripts in air-liquid interface-differentiated bronchial epithelial cells (BECs). These investigators concluded that CS exposure significantly decreased CFLAR expression in BECs. Furthermore, there was a shift in relative levels of the isoform c-FLIP_S_ and c-FLIP_L_ isoform transcripts in bronchial biopsies of smokers compared to non-smokers, correlated with cell death. The proof of concept came from *in vitro* downregulation of CFLAR by siRNA, which showed increased apoptosis, necrosis, and DAMP release as observed in CS. As this study concluded, CS exposure downregulates CFLAR expression. Moreover, decrease in c- FLIP might increase susceptibility of BECs to immunogenic cell death. Therefore, c-FLIP serves as a molecular biomarker for DAMPs and COPD [127–129].

## Conclusion

Deregulation of c-FLIP plays crucial roles in several diseases including cancer, AD, and COPD. c-FLIP is a master apoptosis regulator frequently overexpressed in various malignancies and its upregulation is often correlated with a poor prognosis. It is well documented that c-FLIP isoforms induce resistance to death receptor ligands such as TRAIL, chemotherapeutic agents, and various cell death mechanisms in malignant cells, and is a rational target for modulating therapy-resistant human malignances Transitory pharmacological inhibition of c-FLIP is adequate to sensitize cancer cells to chemotherapeutics. Agents that indirectly target c-FLIP, such as the drug entinostat (also known as MS-275 or SNDX-275) used for aromatase inhibitor-resistant breast cancer, have a therapeutic window and usefulness which suggests more specific c-FLIP-targeted agents will also be tolerated and effective. Moreover, in addition to high expression of c-FLIP in various cancers, specific mutations may serve as biomarkers and provide c-FLIP “dependency” in certain cancers. For example, an important function of c-FLIP_L_ is activation of the NLRP3 and AIM2 inflammasomes and NSCLC cells with NLRP3 mutations are hypersensitive to cell death due to the loss of c-FLIP expression. Therefore, this hypersensitivity serves as a potential biomarker to identify a subgroup of patients who display enhanced vulnerability to c-FLIP-targeted therapies. Significantly, c-FLIP plays major roles in promoting the survival of immunosuppressive tumor promoting immune cells and function in inflammation, Alzheimer’s disease and COPD. Thus, the foregoing discussion and these conclusions show that c-FLIP can serve as a versatile biomarker for cancer prognosis, a diagnostic marker for several diseases, and as a therapeutic window for direct targeting.

## Abbreviations:

FADD: Fas-Associated *Via* Death Domain
c-FLIP: Cellular FLICE (FADD-Like IL-1β-Converting Enzyme)-Inhibitory Protein
AD: Alzheimer’s Disease
COPD: Chronic Obstructive Pulmonary Disease
UPR: Unfolded Protein Response
